# Assessment of the Relationship between Monocyte to High-Density Lipoprotein Ratio and Myocardial Bridge

**DOI:** 10.5935/abc.20180253

**Published:** 2019-01

**Authors:** Asim Enhos, Kahraman Cosansu, Mustafa Ahmet Huyut, Fahrettin Turna, Erdem Karacop, Nijad Bakshaliyev, Aydin Nadir, Ramazan Ozdemir, Mahmut Uluganyan

**Affiliations:** 1 Bezmialem University, Istanbul - Turkey; 2 Sakarya Educational and Research Hospital, Istanbul - Turkey; 3 Bezmialem University, Istanbul - Turkey

**Keywords:** Biomarkers/blood, Cholesterol, HDL/blood, Monocytes/citology, Myocardial Bridging, Atherosclerosis, Inflammation

## Abstract

**Background:**

Assessing the monocyte to high-density lipoprotein ratio (MHR) is a new tool for predicting inflamation, which plays a major role in atherosclerosis. Myocardial bridge (MB) is thought to be a benign condition with development of atherosclerosis, particularly at the proximal segment of the brigde.

**Objective:**

To evaluate the relationhip between MHR and the presence of MB.

**Methods:**

We consecutively scanned patients referred for coronary angiography between January 2013- December 2016, and a total of 160 patients who had a MB and normal coronary artery were enrolled in the study. The patients’ angiographic, demographic and clinic characteristics of the patients were reviewed from medical records. Monocytes and HDL-cholesterols were measured via complete blood count. MHR was calculated as the ratio of the absolute monocyte count to the HDL-cholesterol value. MHR values were divided into three tertiles as follows: lower (8.25 ± 1.61), moderate (13.11 ± 1.46), and higher (21.21 ± 4.30) tertile. A p-value of < 0.05 was considered significant.

**Results:**

MHR was significantly higher in the MB group compared to the control group with normal coronary arteries. We found the frequency of MB (p = 0.002) to increase as the MHR tertiles rose. The Monocyte-HDL ratio with a cut-point of 13.35 had 59% sensitivity and 65.0% specificity (ROC area under curve: 0.687, 95% CI: 0.606-0.769, p < 0.001) in accurately predicting a MB diagnosis. In the multivariate analysis, MHR (p = 0.013) was found to be a significant independent predictor of the presence of MB, after adjusting for other risk factors.

**Conclusion:**

The present study revealed a significant correlation between MHR and MB.

## Introduction

Myocardial bridge (MB), which was described early in the cardivascular literature, is an anatomical variation characterized by the narrowing of some of the epicardial coronary arterial segments during systole. MB, also known as muscular bridge, is a rare congenital disease with a relatively good prognosis.^1^^-^^3^ It has an estimated frequency of 0.5-2.5% in angiographic series, and it frequently involves the left anterior descending artery.^1^ Although it is considered a benign anomaly, it may lead to complications such as angina pectoris, acute myocardial infarction, coronary spasm, arrhythmias, syncope, and sudden cardiac death.^4^^,^^5^ Systolic compression of the epicardial artery is visible on angiographic imaging. Diagnosis can be made using quantitative angiography, intracoronary ultrasound, or Doppler flow measurement.^6^^-^^8^

Monocyte activation has been known to play an important role in chronic inflammation and cardiovascular disease, in which monocytes and differentiated macrophages can modulate inflammatory cytokines.^9^ HDL is highly effective at inhibiting the endothelial expression of adhesion molecules and preventing monocyte recruitment to the artery wall.^9^ Therefore, while monocytes exert a proinflammatory effect, HDL functions as a reversal factor during this process. Monocyte to HDL-cholesterol ratio (MHR) is a simple assessment method for inflammatory status.^10^ MHR has also been reported as a new prognostic marker in cardiovascular diseases.

It is known that atherosclerosis is an inflammatory process and that MHR is a simple tool for assessing proinflamatory status.^9^^,^^10^ Atherosclerosis has been shown to develop especially at the proximal and distal segments of MB in most patients.^11^^-^^13^ In the present study, we evaluate the association between MHR and MB.

## Methods

### Study Population

We consecutively scanned patients referred for coronary angiography between January 2013- December 2016, and a total of 160 patients who had a MB and normal coronary artery were enrolled in the study. The patients’ angiographic, demographic and clinic characteristics of the patients were reviewed from medical records. Patients with acute coronary syndrome, previous cardiac surgery, known coronary artery disease, concomitant valvular disease, cardiomyopathy, heart failure, atrial fibrillation, congenital heart defects, renal or hepatic disease, malignancy, hematological disorders, and acute or chronic inflammatory disorders were excluded from this study. The study was approved by the local ethics committee.

### Angiographic analysis

Coronary angiography was performed using the standard Judkins’ technique with a biplane cineangiography system. Coronary arteries in the left and right oblique planes and in the cranial and caudal angles were demonstrated. Iopromide (Ultravist-370; Schering AG, Berlin, Germany) was used as the contrast agent, and it was manually injected (4-6 ml of contrast agent in each position) during the coronary arteriography. All of the angiograms were evaluated by two experienced physicians. The presence of MB was defined according to the following criteria: narrowing of coronary vessel lumen during systole and dilation during diastole; no evidence of coronary vasospasm. Based on the findings of coronary angiography, the patients were divided in two subgroups: group A (n = 84) with normal coronary arteries; and group B (n = 76) with MB.

### Laboratory measurements

Blood sample was collected from the antecubital vein using a 21-gauge sterile syringe in laboratory. Monocytes and HDL-cholesterols were measured via complete blood count. MHR was calculated as the ratio of the absolute monocyte count to the HDL-cholesterol value.

### Statistical analysis

All the statistical data were analyzed using SPSS 15.0 for Windows (SPSS Inc., Chicago, IL, USA). Continuous data were expressed as mean ± standard deviation, and the categorical data were expressed as percentages. Continuous variables were tested for normal distribution using Kolmogorov-Smirnov test. Both groups were compared using chi-square test or Fisher’s exact test for qualitative variables when appropriate, and independent t-test for normally distributed continuous variables. The non-normally distributed continuous variables are presented as median and interquantile range. Pearson test was used in the correlation analysis between parametric variables. Receiver-operating characteristic (ROC) analysis was performed for MHR in order to determine optimal cut-off values and to obtain the sensitivity and specificity for each variable to predict the presence of MB. A multivariate logistic regression model was performed by including the parameters that differed significantly between the groups in order to identify the independent predictor of patients with MB. A p-value of < 0.05 was considered significant.

## Results

Seventy-six MB (mean age: 52.3 ± 11.7 years, 82.0% male) and 84 age- and gender-matched control participants with normal coronary arteries (mean age: 53.8 ± 12.2 years, 75.0% male) were enrolled in this study.

Both groups’ baseline demographics, as well as their clinic and laboratory characteristics, are summarized in Table 1. Diabetes mellitus and smoking were found to be lower in the MB group compared to the control group. There was no difference between two groups in terms of other demographic or clinic findings. When laboratory parameters were compared, creatinine, white blood cell and neutrophil were significantly higher in the MB group compared to the control group. However, HDL and total cholesterol were found to be significantly lower in the MB patients. Moreover, the monocyte/HDL ratio was found to be significantly higher in the MB group compared to the control group. The remaining laboratory parameters did not differ between both groups.

**Table 1:** Demographic, clinic and laboratory characteristics of the groups studied

Variables	Control	Myocardial bridge	p value
Age in years	53.8 ± 12.2	52.3 ± 11.7	0.435
Male gender, n(%)	63(%75)	62(%82)	0.315
Hypertension, n(%)	32(%38)	19(%25)	0.076
Diabetes mellitus, n(%)	18(%21)	6(%8)	0.017
Smoker, n(%)	36(%43)	19(%25)	0.018
Glucose, mg/dl	104 ± 23	97 ± 13	0.088
Creatinine, mg/dl	0.83 ± 0.18	0.95 ± 0.72	0.035
Hemoglobin, gr/dl	13.8 ± 1.8	14.3 ± 1.7	0.077
White blood cell count, x 10^3^/L	7.4 ± 1.8	8.2 ± 2.1	0.018
Neutrophil count, x 10^3^/L	4.28 ± 1.42	4.81 ± 1.57	0.021
Lymphocyte count x 10^3^/L	2.31 ± 0.89	2.44 ± 0.75	0.121
Monocyte count x 10^3^/L	0.56 ± 0.15	0.62 ± 0.21	0.149
RDW	14.4 ± 1.7	14.9 ± 1.6	0.060
PDW	15.2 ± 3.2	17.1 ± 2.9	< 0.001
Platelet count x 10^3^/L	238 ± 59	255 ± 76	0.222
LDL cholesterol, mg/dl	123 ± 32	117 ± 27	0.168
HDL cholesterol, mg/dl	49 ± 12	39 ± 8	< 0.001
TG, mg/dl	152 ± 103	136 ± 54	0.909
Total cholesterol, mg/dl	200 ± 48	186 ± 32	0.021
MHR	12.20 ± 4.87	16.31 ± 6.47	< 0.001

RDW: red cell distribution width; PDW: platelet distribution width; HDL: high density lipoprotein; LDL: low density lipoprotein; TG: triglyceride; MHR: mononcyte count/HDL cholesterol ratio.

MHR values were divided into three tertiles as follows: lower (8.25 ± 1.61); moderate (13.11 ± 1.46); and higher (21.21 ± 4.30) tertile (Table 2). We found the frequency of MB (p = 0.002), male gender (p = 0.04) and the WBC count (p < 0.001) to increase as the MHR tertiles rose.

**Table 2:** Demographic, clinic and laboratory characteristics of the MHR tertiles

Variables	1^st^ tertile (n:54)	2^nd^ tertile (n:53)	3^rd^ tertile (n:53)	p-value
MHR	8.25 ± 1.61	13.11 ± 1.46	21.21 ± 4.30	< 0.001
NLR	2.10 ± 1.35	1.98 ± 0.96	2.31 ± 1.16	0.332
Myocardial bridge, n(%)	16(%30)	26(%49)	34(%64)	0.002
Male gender, n(%)	37(%69)	41(%77)	47(%88)	0.041
Hypertension, n(%)	20(%37)	16(%30)	15(%28)	0.593
Diabetes mellitus, n(%)	8(%15)	10(%19)	6(%11)	0.553
Smoker, n(%)	13(%24)	19(%36)	23(%43)	0.105
Age	56 ± 11	55 ± 10	49 ± 14	0.006
White blood cell count, x 10^3^/L	6.80 ± 1.63	7.80 ± 1.99	8.72 ± 1.88	< 0.001
Hemoglobin, gr/dl	13.5 ± 1.8	14.1 ± 1.5	14.5 ± 1.8	0.011
RDW	14.6 ± 1.9	14.6 ± 1.4	14.7 ± 1.6	0.973
Platelet count x 10^3^/L	250 ± 65	240 ± 76	248 ± 63	0.739
PDW	9.2 ± 1.6	9.1 ± 1.5	9.1 ± 1.6	0.940
Glucose, mg/dl	100 ± 15	101 ± 21	101 ± 21	0.964
Creatinine, mg/dl	0.84 ± 0.17	0.85 ± 0.18	0.86 ± 0.16	0.703
LDL cholesterol, mg/dl	127 ± 31	121 ± 29	111 ± 27	0.020
HDL cholesterol, mg/dl	53 ± 11	43 ± 8	37 ± 8	< 0.001
TG, mg/dl	123 ± 47	153 ± 88	159 ± 104	0.060
Total cholesterol, mg/dl	204 ± 40	197 ± 46	179 ± 34	0.004

RDW: red cell distribution width; PDW: platelet distribution width; MHR: Mononcyte count/HDL cholesterol ratio; NLR: neutrophil / lymphocyte ratio; TG: triglyceride.

**Figure 1:**
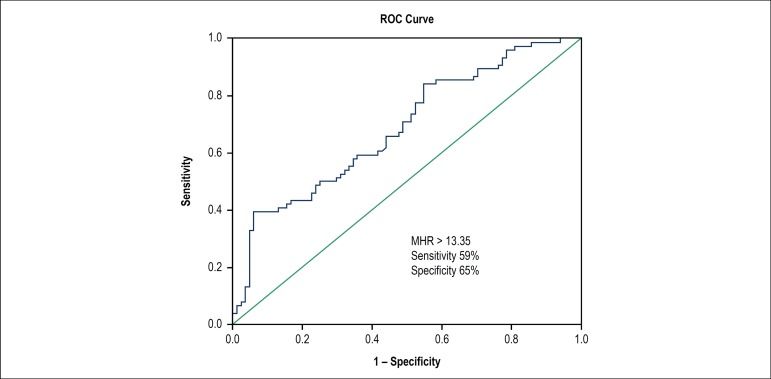
The receiver operative characteristic curve analysis of monocyte to high density lipoprotein cholesterol rate for predicting the presence of myocardial bridge.

A receiver operating curve (ROC) was generated for sensitivity and specificity, with the respective areas under the curve (AUC), to investigate the predictive value of monocyte/HDL ratio for the presence of MB (Figure 1). The Monocyte/HDL ratio with a cut-point of 13.35 had 59.0% sensitivity and 65.0% specificity (ROC area under curve: 0.687, 95% CI: 0.606-0.769, p < 0.001) in accurately predicting MB diagnosis.

In a univariate regression analysis, age, gender, total cholesterol, neutrophil to lymphocyte ratio (NLR), and hemoglobin were significantly related with MB. In the multivariate analysis, MHR (p = 0.013) was found to be significant as the independent predictor of MB, after adjusting for other risk factors (Table 3).

**Table 3:** Multivariate analysis to detect independent variables for the diagnosis of myocardial bridge

Variables	Odds ratio	Confidence İnterval(%95)	p-value
Age	1.010	0.979 - 1.041	0.540
Gender	1.273	0.463 ­- 3.494	0.640
Total cholesterol	0.995	0.986 - 1.004	0.288
MHR	1.128	1.055 - 1.207	< 0.001
NLR	1.012	0.750 - 1.367	0.936
Hemoglobin	1.145	0.896 - 1.463	0.278

MHR: Mononcyte count/HDL cholesterol ratio; NLR: neutrophil / lymphocyte ratio.

## Discussion

The main findings of the present study were as follows: 1) A raised monocyte/HDL ratio was found to be significantly higher in patients with MB; 2) The monocyte/HDL ratio with a cut-point of 13.35 had moderate sensitivity and specifity to diagnose MB; and 3) MHR was found to be a significant independent predictor for presence of MB, after adjusting for other risk factors in multivariate analysis.

Myocardial bridging, which is the compression of a coronary artery segment during systole, is generally accepted to be clinically benign, but it can result in a wide clinical spectrum, from angina to myocardial infarction.^12^^,^^14^^-^^16^ In general, the coronary vessel segment proximal to the bridge has been reported to develop atherosclerosis at an increased rate - up to 90%.^12^^,^^14^ However, one study has also demonstrated diffuse intimal thickening in the tunneled segment.^16^ Besides the tunneled and proximal artery segments, other parts of the same coronary artery, as well as different arteries, could show atheroslerosis.^16^ Endothelial cell morphology variations occur before and after tunneled segment due to blood flow shear stress.^1^ Endothelial dysfunction, inflammation and unknown increased expression of vasoactive agents, such as endothelial nitric oxide synthase, endothelin-1, and angiotensin, all of which convert enzyme in the proximal segment of the MB artery, are the main pathophysiological mechanisms for increased atherosclerotic plaque formation.^13^^,^^17^ Coronary angiography, intracoronary doppler ultrasonography, intravascular ultrasound, fractional flow reserve and cardiac computed tomography angiography are main tools for diagnosing coronary MB.^18^

Monocytes are a source of various cytokines and molecules that interact with endothelial cells, which leads to an aggravation of inflammatory pathways.^19^ Inflamation play a major role in atherosclerosis development and progression.^10^ HDL cholesterol, which has antiinflammatory, antioxidant, and antithrombotic properties, strongly decreases the endothelial expression of adhesion molecules and prevents monocyte recruitment to the artery wall.^20^ Furthermore, HDL decrease pro-inflammatory and pro-oxidant effects of monocytes by inhibiting the migration of macrophages and the oxidation of the low-density lipoprotein (LDL) molecules, as well as by promoting the efflux of cholesterol from these cells.^21^ Therefore, it seems logical to combine these two parameters into a single ratio as an MHR, which can reflect the underlying inflammation process. A prognostic value of MHR has been reported in various cardiovascular diseases.^22^^-^^24^ MHR was found to be related with major cardiovascular adverse events (MACE) including stent thrombosis and mortality after primary percutaneous coronary intervention (PCI) in ST-segment elevation myocardial infarction (STEMI) patients.^25^ Moreover, it has been demonstrated to be a new potential marker for predicting bare metal stent restenosis.^26^ An important association between pre-procedural MHR levels and atrial fibrillation recurrence after ablation procedures was demonstrated by the study of Canbolat et al.^24^ MHR is alwo well demontrated to be associated with coronary slow flow and coronary actesia, which are different forms of inflammation and atherosclerosis.^10^^,^^27^ Our study has reported, for the first time, an important relationship between admission MHR and the presence of MB. Moreover, and concordant with previous studies on various cardiovascular diseases, MHR was found to be a significant independent marker associated with MB, with moderate sensitivity and specifity.

The main pathophysiological links between MHR and MB can be endothelial dysfunction and inflammation. Inflammation not only leads to monocyte secretion and aggregation, but it also reduces HDL blood levels and its anti-oxidative feature.^10^ Increased MHR was associated with systemic inflammation and endothelial dysfunction, and it was defined as a novel inflammation-based prognostic marker in cardiovascular diseases.^22^^-^^24^ In our study, concordant with previous studies on cardiovascular disease, increased MHR was found to be related with the presence of MB, in whose pathophysiology inflammation plays a significant role.

Even though previous studies demonstrated that MHR is associated with systemic inflamation, we found in the present study that MHR is associated with MB. As generally known, a local atherosclerotic process is present in patients with MB, particularly in the proximal and distal segments of the MB. We suppposed that MHR could demonstrate not just systemic artheriosclerosis, but also local artheriosclerosis. With the addition of the local changes at the near of the MB atherosclerosis could be started earlier.

There are some limitations in our study. It was conducted with a small population, and it is a single-center study. Since we measured MHR only at baseline, serial MHR changes were not assessed. A prognostic value of MHR for MB was not determined due to a lack of follow-up of the study patients. Moreover, the effect of other inflamatory markers, like C-reactive protein, was not assesed due to a lack of records.

## Conclusions

In conclusion, since increased MHR is a marker of inflammation and atheroclerosis, MB could be one of the factors associated with increased MHR.
